# The Detection of *Helicobacter hepaticus* Using Whispering-Gallery Mode Microcavity Optical Sensors

**DOI:** 10.3390/bios5030562

**Published:** 2015-08-07

**Authors:** Mark E. Anderson, Emily C. O’Brien, Emily N. Grayek, James K. Hermansen, Heather K. Hunt

**Affiliations:** 1 Department of Biochemistry, University of Missouri, Columbia, MO 65211, USA; E-Mail: meawm4@mail.missouri.edu; 2 Department of Bioengineering, University of Missouri, Columbia, MO 65211, USA; E-Mails: ecoqm9@mail.missouri.edu (E.C.O.); engfg4@mail.missouri.edu (E.N.G.); jkhf34@mail.missouri.edu (J.K.H.)

**Keywords:** *H. hepaticus*, sensors, microcavities, bacterial detection, optical transducing mechanisms

## Abstract

Current bacterial detection techniques are relatively slow, require bulky instrumentation, and usually require some form of specialized training. The gold standard for bacterial detection is culture testing, which can take several days to receive a viable result. Therefore, simpler detection techniques that are both fast and sensitive could greatly improve bacterial detection and identification. Here, we present a new method for the detection of the bacteria *Helicobacter hepaticus* using whispering-gallery mode (WGM) optical microcavity-based sensors. Due to minimal reflection losses and low material adsorption, WGM-based sensors have ultra-high quality factors, resulting in high-sensitivity sensor devices. In this study, we have shown that bacteria can be non-specifically detected using WGM optical microcavity-based sensors. The minimum detection for the device was 1 × 10^4^ cells/mL, and the minimum time of detection was found to be 750 s. Given that a cell density as low as 1 × 10^3^ cells/mL for *Helicobacter hepaticus* can cause infection, the limit of detection shown here would be useful for most levels where *Helicobacter hepaticus* is biologically relevant. This study suggests a new approach for H. hepaticus detection using label-free optical sensors that is faster than, and potentially as sensitive as, standard techniques.

## 1. Introduction

*Helicobacter hepaticus* (*H. hepaticus*) is a bacterium that causes chronic hepatitis and liver cancer in mice [1]. It is a gram-negative, spiral-shaped bacterium of approximate length 2–4 μm that contains a single sheathed flagellum [2]. Like other *Helicobacter* species, *H. hepaticus* is a microaerophillic organism which can survive in acidic environments, such as the mammalian stomach, and can often be found in the mucosal layer of the liver or gastrointestinal tract. Of particular importance to the medical field is that *H. hepaticus* is closely related to the bacterium *Helicobacter pylori* (*H. pylori*) [3]. In terms of genetic similarity, of all the open reading frames in *H. hepaticus*, 938 of them (50.2%) have orthologs in the *H. pylori* genome [1]. *H. pylori* is largely responsible for the development of peptic ulcers, and can be found in the upper gastrointestinal tract of about half of the world’s population [4].

Currently, there are a wide variety of techniques to detect bacteria such as *H. hepaticus*. These techniques include polymerase chain reaction (PCR), enzyme-linked immunosorbent assay (ELISA), infrared light based devices, chemical assays specialized to a specific bacteria (such as the urease breath test for *H. pylori* detection), and culturing, which is still the gold standard for detection [5,6,7,8,9,10,11]. These methods can easily detect between 10 and 100 CFU/mL (for molecular methods), and automated culturing can detect as low as 1 CFU/mL [12]. Although these are proven methods for detecting bacteria, they still have several drawbacks: they can be expensive, they require bulky instrumentation, they necessitate trained personnel to carry out, and as with culturing, they can take multiple days to obtain a result. This study demonstrates an alternative method based on label-free optical detection that can detect bacteria rapidly in a sensitive manner.

Optical detection systems, which rely on the manipulation and measurement of the properties of light that is either transmitted through, absorbed by, or emitted from a sample, have revolutionized medical diagnostics and other monitoring fields [13]. For instance, colorimetric assays, such as measuring the acidity of a substance using pH strips, were among the first optical-based monitoring techniques [14]. More complex methods rely on the analytical measurement of light interacting with a sample in a measurable way, e.g., spectrophotometry. The advantages of using optical systems as the basis for detection platforms are that light is relatively easy to create/manipulate and optical based techniques are often quick and non-destructive to the sample [15]. Optical systems have been used for the detection of materials such as small biomolecules [16], chemical ions [17], and nanoparticles [18].

**Figure 1:**
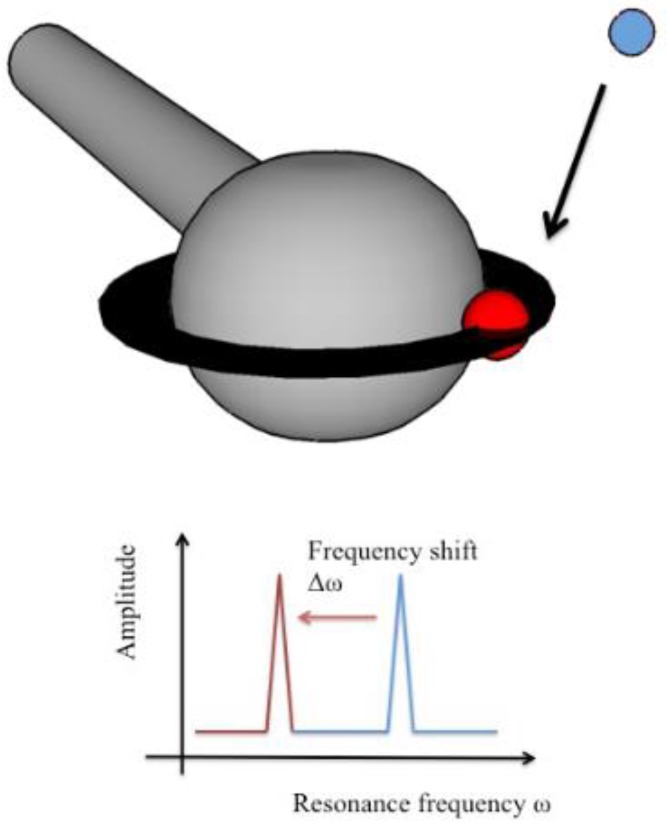
A model for WGM optical microcavity detection, based on [41] (adapted with permission). In the top image, light (thick black line around the device) enters a WGM optical microcavity, where it experiences total internal reflection (TIR) and generates an evanescent field. The evanescent field is an optical field extending to the surrounding environment and decreasing exponentially with the distance away from the resonator’s interface. When an analyte (red sphere), such as bacteria, binds or adsorbs onto the surface of the microsphere, it changes the effective refractive index of the circulating optical field resonator, and it pulls part of the evanescent field to the outside of the resonator. The expansion of the optical field’s boundary causes the round-trip wavelength of light to increase about 2πΔl. The increase in the optical field’s wavelength results in a corresponding frequency shift in the transmission spectrum (bottom image).

Optical sensors are classified into either label-based or label-free devices [19]. Label-based sensors require the analyte to be modified with the addition of a label that is detectable by the sensor. These labels include, but are not limited to, fluorophores, luminophores, enzymes, and nanoparticles [20]. Most detection methods use labeled based detection because label-free detection schemes are often not readily available [21]. Label-free detection systems often have the advantage of being faster (no manipulation of the analyte required) and less intrusive to the analyte [22]. Many label-free optical sensors are based on the refractometric properties of light: that is, they can accurately detect minute changes in the refractive index of an optical field interacting with the analyte. These sensors, which include Surface Plasmon Resonance (SPR) and Whispering Gallery Mode (WGM) optical devices, have become more popular in recent years due to their speed, sensitivity, and label-free sensing properties [7,23,24]. WGM optical microcavity-based sensor platforms utilize an optical microcavity that confines light via total internal reflection around its periphery. There are multiple types of geometries for WGM optical microcavities, such as spheres, toroids, disks, and rings, that have been used for this purpose [25]. Due to their ultra-low loss, based on their silica material system and their nearly atomically smooth, spherical surface, light can circulate around these microspheres. While the light is reflected around the periphery of the device, it is not totally confined, and extends into the surrounding environment through evanescence. This evanescent tail region is capable of interacting with the device surface, as well as the surrounding environment. As the optical field circulates, each pass allows it to interact with the environment, effectively amplifying the sensitivity by the number of circulating loops, which is measured by the photon lifetime in the cavity, or the quality (Q) factor of the device. Microcavities with Q > 100 million result in a photon lifetime of greater than 100 ns [26,27]. Higher photon lifetimes result in a lower limit of detection. During detection, if an analyte adsorbs onto the device’s surface, the circulating optical field experiences a change in the effective refractive index, causing an extremely rapid and detectable shift in the resonant frequency and amplitude of response. This is, ultimately, why WGM optical microcavities are ultra-sensitive and is the basis for their sensing capabilities [28]. Figure 1 illustrates this process of how WGM optical microcavities are used to detect various substances. A small molecule, depicted by a blue sphere, comes into contact with the grey microsphere. This changes the effective refractive index of optical field circulating around the microsphere and thus changes the resonant wavelength of the optical field (seen in black). The change in wavelength, as depicted by the bottom graph, can be tracked over time using a custom-built LabVIEW program.

Research in recent years has shown WGM optical microcavity devices have been used to detect various inorganic materials such as nanoparticles [29,30,31,32], gas [33], and polymers [34], as well as biological substances like proteins [35,36,37,38]. Moreover, they have been shown capable of performing single-molecule detection, depending on the analyte and the device [31,39]. Therefore, biosensor platforms based on these devices could significantly improve upon standard detection techniques in use for bacterial detection, based both on their sensitivity, as well as their fast detection (usually in seconds), particularly when coupled with a microfluidics sample delivery system, as discussed elsewhere [28].

In this experiment, *H. hepaticus* is non-specifically detected in phosphate buffered saline (PBS) using WGM optical microcavity-basedsensors created from silica microspheres that are approximately 200 μm in diameter as the optical microcavities. Previous work has established the mechanism for detection based on the unique binding kinetics of rod-like bacteria on a WGM microcavity. The distinctive kinetics were due to the irregular shape and relatively large size of the bacteria in comparison with other analytes that have been addressed with these types of label-free optical sensors [40]. In this paper, for the first time, we demonstrate the capability of the WGM optical microcavity-based sensor platform for the bacterial detection of *H. hepaticus* at relevant concentrations, and demonstrate that this detection is much faster than standard techniques.

## 2. Experimental Section

### 2.1. Helicobacter hepaticus (H. hepaticus) Preparation

*H. hepaticus* was cultured from a frozen stock in a biosafety level 2 facility. The process was started by adding 3–5 mL of warm brucella broth (BD) to 3 tryptic soy agar with 5% blood plates (Fisher Scientific) under a laminar flow hood. When the *H. hepaticus* had thawed (~1 mL), it was distributed evenly across the three plates. The plates were then placed in a microanerobic pressure chamber (Fisher Scientific) and kept overnight. The chamber was pressurized at 5 psi with a mixed gas content of 4.89% CO_2_ and 5.02% H_2_ with a balance of N_2_. The next day, the plates were removed from the microanerobic chamber. Using a sterile pipette, small amounts of fluid were removed from the plates. Slides were then created to check for contamination. If there was no contamination, the solution containing the bacteria on the plates was pipetted into a 15 mL conical tube for storage and transportation purposes. Once it was ready for use, 10 mL of the *H. hepaticus* solution was vacuum filtered using a 0.45 μm analytical test filter funnel (Fisher Scientific). The filter membrane was then washed with 10 mL of phosphate buffer saline (PBS). After the wash, the cells were stored at 4 °C until they were ready for use.

### 2.2. Microsphere Fabrication

A storage housing chamber for the microspheres was constructed so as to prevent damage of the microspheres during storage. To create this chamber, a 1/4 inch thick piece of cardboard was cut into a 1 inch × 1 inch square using a pair of scissors. This square was then taped onto a regular-sized glass slide and then a 1 inch section of scotch tape, with its ends meeting to form a roll, was attached to the cardboard. The glass slide was then placed in a petri dish [42].

**Figure 2:**
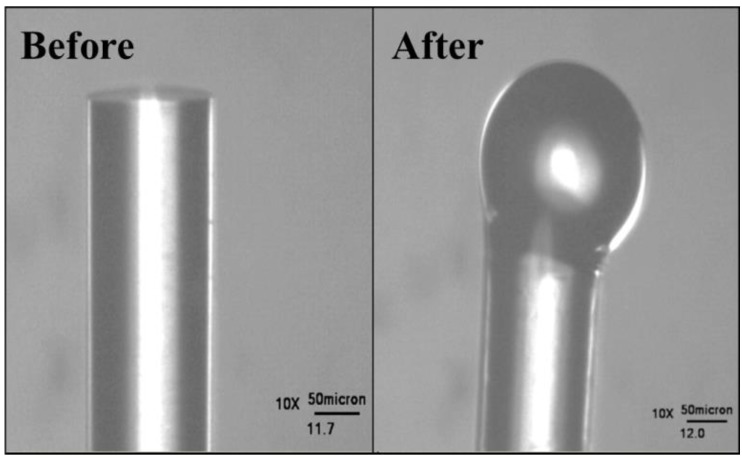
Microscopic images of the tip of a single mode optical fiber before and after gravimetric melting with a CO_2_ laser.

To fabricate the silica microspheres, a 3 inch section of optical fiber from a spool of single mode optical fiber (F-SC, Newport, 980–1550 nm, 0.17–0.19 NA) was cut using a pair of scissors. Then the protective polymeric coating from the last 1/2 inch at the end of the optical fiber was stripped using a No-Nik fiber-stripper. Next, the silica core was exposed by gently wiping the fiber with a Kimwipe that had been dampened with methanol (ACS Grade, Fisher Scientific). After this cleaning, a bare fiber cleaver is used to cut the end of the fiber so that only about 1 millimeter of the stripped fiber remained on the end of the fiber. The stripped end of the optical fiber was placed in the path of a CO_2_ laser (Figure 2).

**Figure 3:**
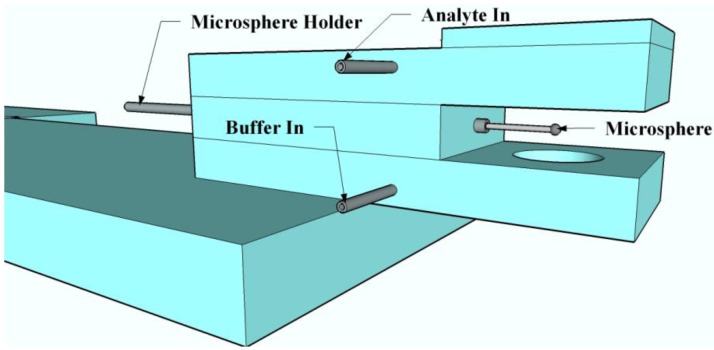
A model of the open-flow flow cell.

A 25 Watt CO_2_ laser (Synrad) was used to gravimetrically melt the tip of a single mode optical fiber into a microsphere. The fiber was vertically aligned so that the stripped end was down. After the fiber was positioned correctly, the laser was turned on, and the laser power was increased slowly to 8.0% power using the laser controller. Once the laser began to melt the tip of the fiber, the fiber was moved vertically (typically less than 0.5 cm) for about 3 s using a stage controller (ThorLabs). After the fiber was moved, the laser is kept on for an additional 3 s. This will cause the laser to gravimetrically melt (Figure 3) the tip of the optical fiber into a sphere, typically with the sphere centered on the optical fiber, but in some cases, as shown, off-center. Any asphericity was taken into account during the device’s use by ensuring that the device was coupled correctly. After the sphere was made, the stem of the microsphere was gripped using tweezers and the sphere was placed into the proper storage housing chamber.

### 2.3. Taper Fabrication

Tapered, single-mode optical fiber was used to couple light from the tunable, CW diode laser into the microsphere device in the under-coupled regime using standard literature procedures. Tapered optical fiber was created by first using a No-Nik fiber-stripper to strip a one inch section of spooled, single mode, optical fiber (F-SC, Newport). The stripped section of fiber is cleaned using a Kimwipe dampened in methanol (ACS Grade, Fisher Scientific). Then, the optical fiber was attached to an optical fiber holder stage (Newport) with the stripped section of fiber in the middle of the stage. The optical fiber holder stage was then placed onto a two-axis stage (Sigma Koki) and the stripped section of optical fiber was found using a 20× microscope (Navitar). Next, a valve to a nearby hydrogen tank, connected to a hydrogen torch (National), was opened. The hydrogen torch was placed directly under the stripped section of the optical fiber. The two-axis stage was controlled from a computer using the SG Commander Program that comes with the Sigma Koki stage. When the hydrogen torch was lit, the SG Commander program was started. This program moved the taper holder stage in the x direction, slowly pulling the stripped section of the optical fiber as it was softened by the hydrogen torch. Using the microscope, the taper waist was monitored, and when the taper waist narrowed to the desired width (~800 nm), the hydrogen valve was closed and the Sigma Koki stage was stopped. The tapered optical fiber was then carefully transported to the resonator setup manually for further use.

### 2.4. Microcavity Characterization

The microspheres were characterized by optical microscopy to determine their geometry and surface quality (Navitar), and then subjected to standard cavity characterization techniques, which resulted in the extraction of the Quality (Q) factor of each sphere (the performance metric for detection) [43]. The Q factor describes the photon lifetime within the microcavity, and should be above 10^6^ (ultra-high-Q regime) to perform high sensitivity detection [44]. To test for the Q factors, light from a CW, tunable diode laser centered at 980 nm (Newport, TLB-6300-LN), controlled by a custom LabView program, was directed through the tapered optical fiber discussed previously, where it then evanescently coupled into the device, and from there traveled into a photodiode detector (Thor Labs, PDA36A). The optical signal was then transmitted into an oscilloscope built-in to the computer system. Resonant wavelengths for each microsphere were then observed on an oscilloscope integrated into the computer system (NI, PCI-5114).

The optical field was evanescently coupled in the undercoupled regime; the under-coupled regime is more favorable than the over-coupled and the critically coupled regimes due to its minimal extrinsic loss [45]. To control the coupling distance and to attain the desired regime, the tapered optical fiber was fixed while the microsphere was placed in a fiber optic positioner (Newport, FP-2A) attached to a three-axis (XYZ) nanopositioning stage (Optosigma) into a position adjacent to the tapered optical fiber. The nanopositioning stage allowed the movement of the microsphere relative to the taper. The appropriate coupling distance was maintained through the use of a three axis fine stage controller (Sigma Koki) for the nanopositioning stage. The device was monitored using side- and top-view cameras simultaneously (Moticam 1000, 1.3M pixel) that were attached to top-view and side-view microscopes (Navitar) positioned around the tapered optical fiber.

A custom LabView program that combined sweep mode, step mode, sensing, and Q factor fit applications was used to obtain the Q factor and sensing data. First, the sweep mode application was used to scan through the entire wavelength range of the laser (975 to 985 nm). Resonances at various wavelengths that were visually representative of a high Q factor were recorded. Next, the step mode application was used to step to those peaks that were marked in the sweep mode scan, and coupling was adjusted to minimize external losses and maximize under-coupling. The recorded wavelength and voltage data were further analyzed using Origin software. The Lorenztian fit of the resonant peak was then used to calculate the linewidth and the Q factor, as shown in Equation (1).

(1) $$Q=\frac{\lambda }{FWHM}$$

The Q factor equals the resonant wavelength, λ, divided by the linewidth, which is the full width at half maximum (FWHM) of the resonance. Throughout the experiments, low input power, in addition to appropriate coupling, were used to minimize non-linear effects during detection. Scan rate and scan speed were held constant during the experiments.

### 2.5. General Sensing Experimental Setup

*H. hepaticus* sensing experiments were carried out with the use of a created open-flow cell (Figure 3). The flow-cell was created out of two pieces of glass with metal tubing embedded at appropriate locations for the injection of the buffer and the analyte solutions. The injected solutions were held within the chamber via surface tension [43]. The microsphere-taper system was first immersed in PBS buffer. Then, PBS containing *H. hepaticus* was pumped into the apparatus using a syringe. When the *H. hepaticus* bacteria interacted with the surface of the microsphere, the microsphere’s resonant frequency changed, as measured by the custom LabView program previously discussed. We note that previous literature has shown no significant resonant peak shift for control experiments completed using only PBS as the analyte [43]. Several different concentrations of *H. hepaticus* in PBS were used to determine the time to detection and the limit of detection with these devices (1 × 10^6^, 1 × 10^5^, and 1 × 10^4^ cells/mL). The sensing experiment consisted of detecting the highest concentration to the lowest concentration of *H. hepaticus* with a single microcavity, with a PBS rinse in between each concentration. The PBS rinse consisted of dipping the microcavity in a solution of fresh PBS and placing the solution containing the microcavity on a rocker tray for 2 min. Then, the sphere was removed, dried, and re-rinsed using the same procedures. Between each concentration run, the microaquarium was cleaned via flushing with PBS to remove any potential contamination in the microaquarium. The entire sensing experiment was repeated four times, each time with a new microcavity to verify the consistency of detection across multiple devices. Note also that the same microcavity size was used for all repeats to minimize the impact of a change in the microcavity size on the wavelength shift.

### 2.6. Fine-Tuning the Injection Process

A syringe pump was used to inject analyte into the system at varying flow conditions. These conditions were based on the maximum volume that the flow cell can hold, the amount of buffer needed to completely immerse the microsphere, and the maximum rate at which solution can be injected into the flow cell without the tapered optical fiber breaking. The flow conditions were 0.010, 0.030, and 0.050 mL/min for the injection rate, and 0.020 and 0.040 mL for the injection volume. It was found that a flow rate of 0.030 mL/min and a volume of 0.040 mL gave the best flow conditions. That is, these were the fastest conditions where the background noise was effectively reduced. This background noise is a product of the microsphere physically moving during the injection process due to induced turbulence. These were the flow conditions used for the entirety of the experiment.

### 2.7. Finding the Limit of Detection and Time to Detection

The filtered *H.hepaticus* and the PBS buffer were warmed to room temperature before sensing. The glass flow cell was cleaned with DI water and allowed to dry at room temperature before each use. A microsphere was positioned in the metal tubing in the glass flow cell. Two syringes, one containing the bacteria and the other PBS buffer, were used to inject the solutions into the flow cell. The syringes were placed in automated syringe pumps (from CHEMYX Inc. and Braintree Scientific Inc.) and the syringes were connected to the flow cell by about three feet of plastic tubing (Tygon Flexible Plastic Tubing). Before connecting the tubing to the flow cell, the solution in the syringe was slowly pumped out until there was a bubble-free solution at the end of the tubing. The diameter of the syringe, the volume of the injection, and the rate at which the injection occurs was programmed into the injector before use. In this study, we used a 5 mL syringe (BD Plastic 5 mL syringe with Luer-Lok Tip) for the buffer and a 3 mL syringe (BD Plastic 3 mL syringe with Luer-Lok Tip) for the analyte. The best volume/rate for the buffer and analyte were fine-tuned as described in the previous section. The glass flow cell that was used in this study could hold a maximum value of 650 μL.

The flow cell was then fastened with screws onto a nanometer based xyz stage. Then the XYZ stage was used to carefully position the microsphere in the flow cell directly underneath the tapered optical fiber. The sphere was then coupled to the tapered optical fiber. 0.300 mL of the PBS Buffer solution was then pumped into the flow cell at a rate of 0.05 mL/min. After the buffer was injected, the coupling of the microsphere and the taper was readjusted as appropriate, until proper coupling was achieved. Then, the syringe pump was then used to inject a known concentration of *H. hepaticus* into the flow cell. Solutions of *H. hepaticus* were created so that the concentrations of the bacteria would be 1 × 10^6^, 1 × 10^5^, and 1 × 10^4^ cells/mL PBS. During each experiment, the shift of the resonance was tracked before, during, and after injection using a custom LabVieW program that contains a built-in oscilloscope. The data was saved as a .txt file and analyzed in the software program Origin. The resulting data represent a function of the shift in wavelength over time.

## 3. Results and Discussion

### 3.1. Culturing H. hepaticus

Cell densities of the *H. hepaticus* cultures were calculated via serial dilutions on a cell plate. The average cell density for the culture was found to be 1 × 10^6^ cells/mL. Once cultured, the bacteria were stored for further use at 4 °C. For every trial, the bacteria were used within 3 days of being cultured.

### 3.2. Device Sensitivity

**Figure 4:**
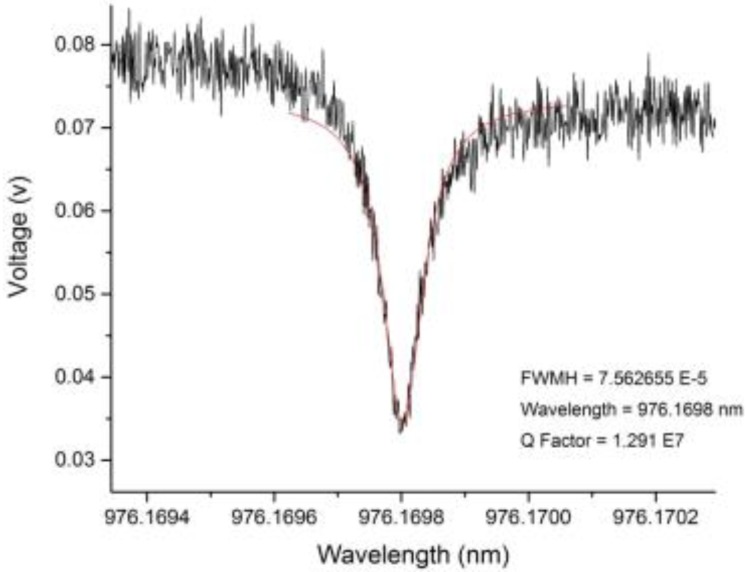
A representative resonance peak of the silica microspheres used as the WGM optical microcavities in the sensing experiments, showing a high quality factor device (black line—data, red line—Lorentzian fit) during testing in air.

The sensitivity of the microspheres was measured by way of quality (Q) factors. In Figure 4, a representative transmission spectrum is shown in black with Lorentzian fit shown in red. This was obtained from raw oscilloscope data of an on-resonance microsphere. For use in a biologically relevant settings, and to be capable of detection of single virus and nanoparticles, Q factors above 10^6^ were needed [47,48,49]. The Q factors for every microsphere used in this study was above 10^7^.

### 3.3. Sensing

**Figure 5:**
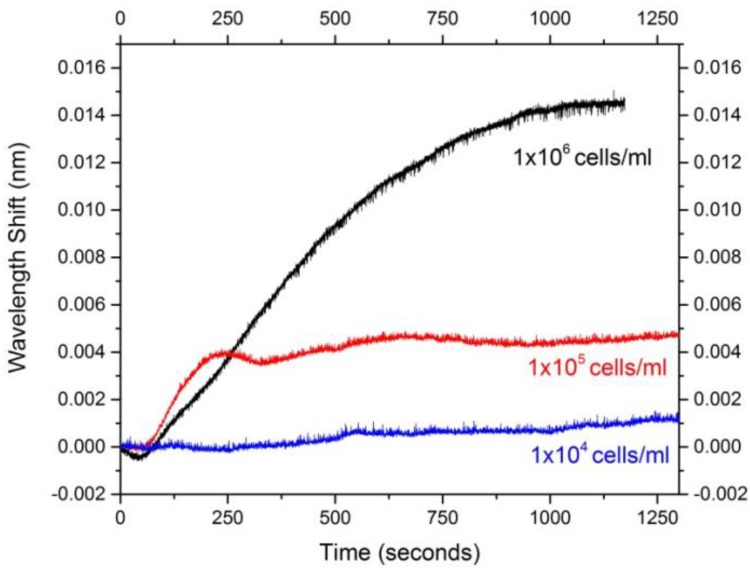
An overlay of the wavelength shift over time for a single representative silica microsphere (sphere 4, referenced in Table 1 and Table 2) used in the sensing experiments.

For detecting *H. hepaticus* with bare silica microspheres, the limit of detection was found to be 1 × 10^4^ cells/mL. As a reference, the lowest cell density that *H. hepaticus* is infectious is around 1 × 10^3^ cells/mL. The detection profile for a sphere is shown in Figure 5. For each sphere at each cell density, the average wavelength shift was calculated (Table 1). The cell densities of *H. hepaticus* that were tested were: 1 × 10^6^, 1 × 10^5^, and 1 × 10^4^ cells/mL in PBS. The average time to detection was also calculated for each sphere at each cell density (Table 2). The times ranged from 500 to 1100 s, with the minimum time for detection at 1 × 10^4^ cells/mL being 750 s. Because the detection time depends on how quickly the bacteria move from the injection point to the sensor surface, the detection time in this case is limited by the extrinsic mass transfer, rather than the sensor itself. For instance, a smaller microaquarium or a faster injection rate would likely significantly decrease the time to detection. This implies that these sensors, when coupled with an appropriate microfluidic delivery system, could provide enhanced detection capabilities.

**Table 1:** Wavelength shift seen for each cell density tested. Wavelength shift correlates to concentration. The Q factors of the spheres, in air, just prior to the beginning of the sensing experiments, are also given.

	Wavelength Shift (pm)							
		*Sphere 1*	*Sphere 2*	*Sphere 3*	*Sphere 4*	*Sphere 5*	*Average Shift*	*Standard Deviation*
**Q factor**		1.19 × 10^7^	1.13 × 10^7^	1.03 × 10^7^	1.04 × 10^7^	2.28 × 10^7^		
**Cell Density (cells/mL)**	1 × 10^6^	25.13	20.78	30.19	14.7	29.81	24.12	6.52
	1 × 10^5^	6.16	6.82	3.34	4.67	4.30	5.06	1.41
	1 × 10^4^	0.22	2.26	1.57	1.14	1.01	1.24	0.75

**Table 2:** Time to detection for each cell density tested, as defined by the amount of time required for the wavelength shift to equilibrate.

Time to Detection (s)							
Cell Density (cells/mL)	*Sphere 1*	*Sphere 2*	*Sphere 3*	*Sphere 4*	*Sphere 5*	*Average Shift*	*Standard Deviation*
1 × 10^6^	550	475	1100	900	750	755	255
1 × 10^5^	1000	900	500	750	700	770	192
1 × 10^4^	1100	1050	1100	1050	750	1010	147

Sensing profiles for one of five microspheres is shown for *H. hepaticus* concentrations of 1 × 10^6^ (black), 1 × 10^5^ (red), and 1 × 10^4^ (blue) cells/mL. The flow cell was buffered in PBS and the bacteria were filtered in PBS. The injection of the bacteria takes place over the course of the first 90 s in the graph. Note that both adsorption and desorption processes can occur during sensing. The curves shown here represent the equilibrium between these processes, rather than complete saturation of the sensor surface by the bacteria. [50] Of particular interest is the significantly larger wavelength shift between the 1 × 10^5^ and 1 × 10^6^ concentration curves. Here, the adsorption process is driven by the large concentration gradient between the solution and the surface, and the higher concentration results in a higher equilibrium amount of bacteria adsorbing on the surface. Because the resulting wavelength shift will depend on the size of the microcavities used, microspheres of the same size (200 μm) were used throughout the study. The minimum detectable concentration of *H. hepaticus* for 200 μm microspheres was found to be 1 × 10^4^ cells/mL, which is the equivalent of 400 cells in the microaquarium solution, based on the volume injected. In comparison, automated culturing techniques can detect down to 1 CFU/mL (live cells), and molecular methods, like PCR, can detect 10–100 CFU/mL (live cells) [12]. Although this technique does not reach the same ultra-low detection limit as alternative techniques, it is still capable of detecting medically relevant levels of *H. hepaticus*.

## 4. Conclusions

This study establishes a proof of concept for the detection of *H. hepaticus* using a WGM optical microcavity-based sensor platform. The minimum detection of 1 × 10^4^ cells/mL demonstrates that WGM based sensors could be used to detect *H. hepaticus* at most medically relevant concentrations. The time of detection, which ranges from 500 to 1100 s, shows that the device works more quickly than most current methods, which typically take hours (e.g., ELISA assays) to days (e.g., culture testing) to complete. The limit of detection for this device could be improved through the use of higher-quality microspheres, as it is well-known that these devices are capable, in certain circumstances, of single-molecule detection. Another potential source of improvement could be implementing different wavelengths for the detection process. For this study, all experiments were completed at 980 nm, so it could prove useful to look into whether different laser sources cause noticeable differences. It is important to note that this study was completed using non-functionalized microspheres, which contain no element of specificity towards *H. hepaticus*. Detection was confirmed because the sensing tests were completed in a simple environment where the bacteria were filtered into a solution of PBS.

For the device to be applicable in a medical setting, the sensor needs to be able to detect bacteria in a complex environment such as wastewater or blood. Future studies will aim to solve this problem by adding an element of specificity to the microspheres. By demonstrating that WGM-based sensors are a viable method for the detection of biological substances, this study lays the groundwork for improved sensors that have the ability to revolutionize the world’s environmental/medical diagnostic equipment in the future.
